# The role of revelation in the revelation effect in episodic recognition

**DOI:** 10.3758/s13421-025-01756-3

**Published:** 2026-05-28

**Authors:** Zehra F. Peynircioğlu, Sara Wong, Joshua R. Tatz

**Affiliations:** 1 Department of Psychology, American University, Washington, DC 20016 USA; 2 Department of Psychology, UCLA, Los Angeles, CA USA; 3 Psychological and Brain Sciences, University of Iowa, Iowa City, IA USA

**Keywords:** Revelation effect, Recognition memory, Aha, Emotion/arousal

## Abstract

Solving a task right before a memory decision tends to elicit a “yes” response in recognition tests, and the underlying mechanism of this so-called revelation effect is still not clear. We suggest that an “aha” moment experienced while solving the task may be an integral factor in promoting the effect, and test this idea in two experiments. Participants solved an anagram of the test item (Experiment 1) or of an item unrelated to the memory test (Experiment 2), or copied the intended word before each recognition decision. They also reported whether the solution of each anagram had been accompanied by an aha moment or not. A revelation effect emerged in both experiments in that items following solving anagrams were judged to have been recognized more often than those following copying of intended words – there was a shift to a more liberal decision criterion. However, this was true *only* when anagram solutions had been accompanied by an aha moment. We discuss the critical role of the phenomenological experience of an aha moment in eliciting the revelation effect.

**Supplementary Information:**

The online version contains supplementary material available at 10.3758/s13421-025-01756-3.

## Introduction

The revelation effect is a positivity bias that emerges during a recognition test. After a presentation list, if another task immediately precedes the memory judgment for a given test item, participants are more likely to say they recognize that item from the presentation list (e.g., Peynircioğlu & Watkins, 1988). These immediately preceding tasks can comprise a variety of exercises, such as one leading to the discovery of what the item is by rotating its letters or solving its anagram (e.g., Watkins & Peynircioğlu, 1990). Other tasks that can elicit the revelation effect, or this feeling of increased familiarity with the test item during the recognition process, can also include the discovery of an unrelated item (i.e., not part of the memory experiment) (e.g., Westerman & Greene, 1996), or a letter-counting or a synonym-generating task that does not require the discovery of the test item itself (Westerman & Greene, 1998). Reported more than three decades ago and explored widely using different tasks, materials, and different situations (e.g., Bornstein & Neely, 2001; Bornstein & Wilson, 2004; Mulligan, 2007; Peynircioğlu & Tekcan, 1993; Young, et al., 2009), the underlying mechanism of the effect is still somewhat of a mystery. Indeed, in a meta-analysis of the revelation effect, Aßfalg et al. (2017a) concluded that none of the existing theories could explain all the memory findings to date.

Some revelation studies have shown this positivity bias also in judgments outside of episodic recognition, such as when asked for truth ratings involving trivia questions or preference ratings involving brand names (e.g., Bernstein, et al., 2002; Kronlund & Bernstein, 2006). Because such non-episodic judgment findings are currently somewhat mixed with respect to the emergence of the revelation effect (e.g., no effect with word/nonword decisions or decisions on the frequency of general usage of words, shown by Watkins & Peynircioğlu, 1990), we recognize that perhaps different judgments differ in the way they are affected by the revelation task. For instance, revelation tasks may have at least partially different underlying mechanisms that influence different judgments, or they may have the same mechanism but affect different judgments in different degrees or levels, or they may elicit reactivity under certain conditions. Exploring that particular mystery is outside scope of this paper, and, thus, in this study, we focus on and limit our conclusions only to episodic recognition judgments.

In memory research, the revelation effect is a robust phenomenon, and we know many of its boundary conditions, such as its limitation to episodic judgments (e.g., Watkins & Peynircioğlu, 1990), even when these judgments are for self-projected past and future events (Westerman et al., 2017), and to younger adults (Prull et al., 1998). We also know some manipulations can sometimes modulate the revelation effect, such as the difficulty of the revelation task (Aßfalg et al., 2017b), type of encoding tasks (Mulligan, 2007), or whether the judgments are based on recollection or fluency (Cameron & Hockley, 2000), although the level of influence and effect sizes vary greatly from task to task (Aßfalg et al., 2017a).

The main purpose of the present study is to report a new and possibly overarching finding that could promote a greater understanding of the revelation effect in recognition memory. This finding harks back to the idea of “revelation,” this time not in the sense of revealing or discovering during a task preceding the memory judgment, but in the sense of an epiphany as a function of flashing realization or even emotion/arousal experienced. The impetus for the study thus comes from the idea that perhaps participants sometimes experience some sort of an aha moment during solving a task that is embedded into but is separate from the memory judgment, and also have a belief, correctly or incorrectly, that, in cases when a to-be-recognized word is revealed, familiar items are more likely to elicit this aha moment. In turn, such an aha moment results in a greater likelihood of both hits and false alarms during the recognition test and elicits the revelation effect. Further, even in cases when the revelation involves an unrelated filler task (e.g., doing arithmetic, or solving anagrams of words that are not part of the memory experiment per se), the jolt/suddenness experienced in such an aha moment task may have an unwarranted carry-over effect to the ensuing recognition judgment for a different item.

Within the problem-solving literature, there is a distinct memory advantage for solutions of problems attained through insight, or the aha experience (e.g., Auble et al., 1979; Danek et al., 2013; Kizilirmak et al., 2016). One explanation for this advantage is the emotion experienced during the aha solutions (e.g., Seifert et al., 1995) and the finding that emotional events are remembered better than others (e.g., Reisberg & Hertel, 2004), also backed by the interplay between the amygdala and the hippocampus during emotional events (e.g., Kensinger & Schacter, 2008). And, individuals appear to be aware of this emotional memory advantage, as shown by judgment-of-learning ratings (e.g., Hourihan et al., 2017). Finally, this emotion is a pleasant one (e.g., Gick & Lockhart, 1995), and EEG findings show that this rewarding feeling of an aha moment arises immediately rather than as a result of some cognitive appraisal (e.g., Oh et al., 2020).

The revelation effect may then at least partly be due to a mistaken logical inference on the part of participants about “If X then Y” implying also the converse, “If Y then X,” and falling for the fallacy of affirming the consequent (e.g., Hastie & Dawes, 2009). In this situation, X would refer to getting a solution with insight and Y would refer to having better memory for it. To be sure, it may happen that episodically familiar items are indeed more likely to elicit insightful solutions, but to date, there is no supporting evidence for this claim. Because individuals realize that emotional events often have better memory representations, they may also attribute any feeling of an aha moment experienced during the revelation task to the strength of the memory, thus creating the illusion that the item must have been studied. Memory illusions based on mistaken inferences are not rare in the literature. For instance, in the font-size effect, participants think they will remember items presented in larger fonts better but do not actually do so (e.g., Rhodes & Castel, 2008). We suggest that the revelation effect is an illusion also partly based on a mistaken inference, this time an inference perhaps resulting from believing in the converse of the relationship between aha moments and memory – that is, believing that “if X then Y” is true then “if Y then X” must also be true. In addition, to the extent that aha moments produce a fleeting pleasant affect and pleasant affect leads to more positive responding (e.g., Park & Banaji, 2000; cf. George & Dane, 2016), the illusion would be further strengthened.

In some ways, this idea is a revisitation of an important paper that posited the “discovery misattribution” hypothesis, and the results of which also touched on the revelation effect (Dougal & Schooler, 2007). The main theoretical difference is that Dougal and Schooler’s focus was on explaining the discovery misattribution findings and the “solving effect,” and they dismissed the revelation effect as a separate phenomenon from the solving effect they tested. As in the revelation effect, their participants reported recognizing solved anagrams more often than the solutions of unsolved anagrams, and, more importantly for our purposes, solving anagrams with an algorithm did not lead to discovery misattribution. The authors suggested that it could thus be the emotional arousal during self-discovery (rather than via following an algorithm) that was then mistaken for the recollective experience. However, because a similar discovery misattribution did not arise when the solved anagram was not the to-be-recognized target itself (cf. Westerman & Greene, 1996), they concluded that this was not the revelation effect.

Our focus is on the revelation effect, and we ask if insight or an aha experience, suggested to be essential for the solving effect, could also be essential for the revelation effect. Thus, of main interest is whether an aha moment assumed to be present for the discovery misattribution findings is a common denominator and also critical for the revelation effect to emerge in the first place. We test this idea by asking about any aha moments directly. We should note that, based on the traditional usages of the terms insight and aha experience in the literature (cf. Gick & Lockhart, 1995) and on their everyday connotations, we used the two terms interchangeably when instructing our participants. However, the two terms may in fact not be interchangeable. For instance, they may reflect different portions of a single construct, with insight (suddenness of discovery) possibly then eliciting the phenomenological aha moment (cf. Bowden, et al., 2005). Different metacognitive feelings may mediate different underlying components of insight and aha experiences (cf. Skaar & Reber, 2020). They may refer to the more objective and more subjective classifications of the same construct, or with the aha experience referring to the most salient or characteristic phenomenological component of insight (e.g., Danek, et al., 2014). Such conceptual distinctions do not matter with respect to our findings. However, because we asked participants to use the aha experience as an indicator of insight, we will be using the “aha moment” as the term for what we are measuring from here on, possibly with the added benefit of greater face validity with respect to our interest in “revelation.”

Before the main experiments, we conducted two extensive pilot studies, and their methods and relevant results are presented in the Online Supplementary Materials. The main purpose of the first was to see if we also lost the revelation effect with our design and materials when the preceding task was solving an algorithm-based anagram (cf. Dougal & Schooler, 2007). In many anagram-based studies showing the revelation effect, an algorithmic solution has almost always been provided to the participants to ensure that they do discover the intended word. Thus, it was important to first replicate Dougal and Schooler’s results showing this unexpected loss of the effect when participants solved anagrams using the algorithm. It is interesting that in previous studies, to make discovery a relatively simple and uniform task, experimenters often used the same algorithm to work for all solutions, and little attention was paid to whether the participants were solving each anagram on their own or whether they were making full use of the single solution key. It is thus possible that the revelation effect was limited only to the few self-solved anagrams, or, even when the algorithm was used, the switching of letters in the same order every time might have become automatic enough at some point for participants to use the algorithm only partially and then let the word emerge with an accompanying aha experience. To that end, in the first pilot experiment, we also introduced many different algorithms as a further precaution to increase the chances of replicating the loss of the revelation effect. In that way, if an algorithm was needed, participants would be likely to use it fully rather than memorizing it and using it just to start things going, thus precluding even a partial aha experience during the solution. Nevertheless, the aha experiences would still be only an assumption made for the self-solved anagrams. The main purpose of the second pilot experiment was to tweak the materials and the method further, and, more importantly, to see if direct reporting of the aha moments would be feasible/meaningful to the participants and thus could replace the indirect testing of such experiences. The results of both pilot studies were positive (we replicated Dougal and Schooler’s findings and found that reporting of aha moments could be used directly for our purposes), which justified the two main experiments reported here.

In the main study, we showed that an aha moment experienced in solving a task before the actual episodic recognition task did indeed have at least a strong influence on the positivity bias, and was perhaps even crucial for the revelation effect to occur. The task before some of the recognition judgments involved solving anagrams. These anagrams were of the to-be-recognized items themselves in the first and of extra-list items in the second experiment. Extra-list items referred to words that had not been presented, and participants were not asked to make any memory judgments on these items. The recognition rates of words that were preceded by solving anagrams of both types were compared to those of words that were not preceded by such a task. The main difference from typical revelation-effect studies was that participants also indicated whether they had an aha experience while solving each anagram or not. We should acknowledge at this point that, although popular in the problem-solving realm, the use of self-reports as aha-experience measures are largely unvalidated because of their subjective nature dealing with a multidimensional construct (cf. Webb et al., 2018). Nevertheless, a healthy revelation effect emerged in both experiments, but only for those items that were reported to have been accompanied by an aha experience, and not otherwise.

## Experiment 1

### Method

#### Participants

A total of 59 participants were tested until we reached our goal of 40 participants who avoided the exclusion criteria and whose results we could analyze, based on our pilot experiments. All participants were American University students (ages 18–30 years), who received extra credit in their psychology courses or participated for fun. The exclusion criteria were as follows: (1) not following instructions (e.g., skipping of copying words or of writing in anagram solutions); (2) not being able to solve at least 80% of the anagrams, and (3) not having at least 10% and at most 90% aha responses pertaining to the anagrams that were successfully solved. Because of COVID-19 interference, the experiment was started in-person (and ten out of 14 avoided the exclusion criteria) and was moved to a virtual platform, using Qualtrics (and 30 of 45 avoided the exclusion criteria). For participants who were tested virtually, the experimental session was conducted remotely using Zoom. Participants were asked to be in a distraction-free area, use a laptop, and share their screen and computer audio. They were also asked to leave their camera on for the entire procedure in order to ensure engagement with the task. All participants were tested individually. None had participated in either of the pilot experiments. All experiments were approved by the American University Internal Review Board (IRB).

A sensitivity analysis in G*Power (Faul, et al., 2009) based on one group, three measurements, and a correlation of 0.34 (calculated based on the weighted average of the mean correlation across conditions from the present experiment and the related Pilot Experiment 1) indicated that our effective sample size of 40 participants enabled 85% power to detect small to medium effects (*f* = 0.25). Notably, this sample size is intermediate to those included in Pilot Experiments 1 and 2, which had included effective sample sizes of 48 and 32 participants, respectively (for these results, see Online Supplementary Materials).

#### Materials, design, and procedure

A total of 72 five- to seven-letter words were selected from the pilot experiments, which had been originally obtained from sources specifying single-solution anagrams (Gilhooly, 1978; Rawlinson, 1976). There were two study lists, yoked in terms of number of letters in each presented word. Each list comprised 40 words, eight of which were used as primary and recency buffers (and these were the same words in both lists), and 32 of which were the target items. Across two groups of participants, one list comprised the targets and the other the lures during the recognition test. The recognition test comprised 64 items, 32 targets and 32 lures, with 16 of each presented as anagrams and the others as words. Across two subgroups of participants, each item also served equally often in the word and anagram conditions.

For in-person study list presentation, words were printed on a single sheet of paper in two columns, and participants uncovered each word at 2-s intervals by sliding a blank sheet of paper when prompted by the experimenter. For virtual study list presentation, each word appeared one at a time on the screen for 2 s. Participants were told to simply try to remember each word. During the yes/no recognition test, items were presented in the same order to all participants, which was random with respect to target/lure status and word/anagram condition and different from the presentation order for targets.

For in-person recognition testing, items were presented in a booklet form, with each item occupying a single line with different columns for responses. For virtual recognition testing, each item was presented on a different screen and the columns were replaced by separate rows. The test was preceded by detailed instructions, especially about insight and aha moments, emphasizing the feelings of jolt, a pop out or a flashbulb experience, and suddenness of realization. In addition, participants were told that we were using insight and aha moments interchangeably and asked to describe an aha moment in their lives before starting the test (the exact “aha” instructions are in the first footnote,^1^ which were then orally elaborated on). There was also a short practice with the procedure using the primacy and recency buffer items.

The procedural instructions were that they would sometimes see a word and they would need to copy it in the fragment next to it (e.g., PLEASE; P - - - - -) and then make a Yes/No judgment on whether the word had been included in the study list and give a confidence rating between 1 (guess) and 3 (sure).^2^ And sometimes the to-be decided-on word would be disguised in the form of an anagram (and these would be asterisked), and they would need to solve it and write the solution in the fragment next to it before making the recognition judgment (e.g., *PELASE; P - - - - -). They were given up to 45 s for this task.

Then, if participants had indeed solved the anagram, they were asked to put a check mark under either the Immediate (about first 3 s) column or the Later (after about 4 s) column (or row in the case of virtual testing) to indicate the timing of the solution. Although this response was in fact subsidiary to our main purpose, we would like to explain the rationale for asking whether anagrams were solved early or later during the solution process. We thought that we could get additional information because in problem-solving studies aha moments are more likely to be elicited after an impasse (e.g., Moss et al., 2011), and thus we might also predict more aha instances after longer solution times, which in turn would have implications for the magnitudes of the revelation effect in the early and late conditions. Such a finding could also have implications for fluency effects as explanations of the revelation effect. That is, because early and late solution anagrams would reflect differences in processing fluency, one could expect differing strengths in revelation effects as a function of solution times. We should note at this point that we did not see any meaningful differences in terms of solution times and the emergence of the aha experience.

During the remainder of the procedure, if the participants did not solve the anagram, they were to simply leave it and move on to the next item (in virtual testing, moving on was accomplished by self-clicking the “next” button). There were thus no recognition judgments for these items. Finally, in all trials that included an anagram that was actually solved, they were asked to indicate if the anagram solution was accompanied by an aha moment or not. They did this by putting a check mark under the “aha” column (or row in the case of virtual testing).

### Results

The results are summarized in Tables 1 and 2. The data were analyzed in JASP (Love et al., 2019) with repeated-measure ANOVAs and paired *t*-tests (with Holm-Bonferroni correction for post hoc comparisons). While we primarily focus on the results of frequentist tests for statistical inference, we also report Bayes factors for all tests (based on two-tailed Bayesian tests using default non-informative priors in JASP). Following JASP guidelines, we considered BF > 3 to represent moderate evidence and BF > 10 to represent strong evidence.

**Table 1:** Mean percent “Yes” responses (targets and lures combined) in word and anagram conditions in Experiment 1, as well as the same for online versus in-person participants. Standard deviations are given in parentheses

	Word	Immediate anagram		Later anagram		All anagrams	
		Aha	No aha	Aha	No aha	Aha	No aha
**Overall**	**51.4 (11.3)**	74.2 (23.8)	51.6 (24.5)	67.4 (27.9)	38.1 (34.0)	**71.9** **(20.1)**	**49.2** **(19.5)**
Online	**51.1 (11.6)**	76.0 (19.8)	49.9 (27.9)	68.4 (29.5)	42.8 (32.8)	**73.8** **(19.4)**	**48.7** **(21.8)**
In-person	**52.3** **(11.1)**	68.1 (34.8)	56.3 (10.8)	64.9 (24.2)	25.0 (35.7)	**66.4** **(22.2)**	**50.9** **(10.6)**

**Table 2:** d's and Cs in the word and with- and without-aha anagram solution conditions of Experiment 1, as well as mean hits and false alarms

	Word	Aha anagram	No-aha anagram
Hits	0.68 (0.16)	0.81 (0.18)	0.60 (0.30)
FA	0.35 (0.19)	0.56 (0.29)	0.41 (0.26)
d'	0.95 (0.86)	0.82 (1.12)	0.61 (1.65)
C	−0.01 (0.39)	−0.65 (0.85)	−0.06 (0.79)

As mentioned in the Method section, the proportions of early (52.9%) and later (58.6%) solved anagrams did not differ, *t*(39) = 0.741,* p* = 0.463, *d* = 0.12, *BF*_01_ = 4.54; and the revelation effect was strong in both time conditions separately, Early Aha versus Words: *t*(35) = 4.12, *p* < 0.001, *d* = 0.69, *BF*_*10*_ = 121.2and Late Aha versus Words: *t*(34) = 5.34, *p* < 0.001, *d* = 0.90, *BF*_*10*_ = 3196.^3^ Thus, we combined the two time conditions for the main analyses. The main analyses were also conducted on all “yes” responses regardless of whether they were hits or false alarms. Because hits and false alarms show any revelation effects in the same direction, “yes” answers to targets and lures are often combined. Further, although usually a greater revelation effect is observed for false alarms, this potential difference was not of interest to us, as the influence of aha moments was predicted to be in the same direction regardless of target/lure status.

In the main analyses, a one-way repeated-measures ANOVA on words, aha anagrams, and no-aha anagrams was significant, *F*(1.64, 63.81) = 29.10, p < 0.001, ${\upeta }_{p}^{2}$ = 0.43, *BF*_*10*_ = 8.25 × 10^7^. Post hoc comparisons showed no differences between words and no-aha anagrams, *t*(39) = 0.72, *p* = 0.474, *d* = 0.11, *BF*_*01*_ = 4.59, but robust differences between words and aha anagrams, *t*(39) = 7.63, *p* < 0.001, *d* = 1.21, *BF*_*10*_ = 4.08 × 10^6^, as well as between no-aha and aha anagrams, *t*(39) = 5.63, *p* < 0.001, *d* = 0.89, *BF*_*10*_ = 1.03 × 10^4^. Thus, a revelation effect emerged but only for anagrams whose solutions were accompanied by an aha moment. We also looked at the aha responses in more detail, and saw that, of solutions that were given “yes” responses to, 63.9% were accompanied by aha moments, whereas of those that were given “no” responses to, 40.2% were accompanied by aha moments, *t*(39) = 6.05, *p* < 0.001, *d* = 0.96, *BF*_*10*_ = 3.67 × 10^4^, corroborating that aha solutions were more likely to be reported as recognized.

Finally, we examined sensitivity (d') and bias (C) in responding. These were computed using standard formulae (d'= z(hit rate) – z (false alarm rate); C = 1/2 * [z(hit rate) + z(false alarm rate)]). In cases where a hit or a false alarm rate was one or zero, the values 0.99999 or 0.00001 were used instead. The d's did not differ across conditions, *F*(2, 74) = 1.04, *p* = 0.36, ${\upeta }_{p}^{2}$ = 0.03, *BF*_*01*_ = 5.17, whereas the Cs did differ, *F*(1.59, 58.9) = 12.77, *p* < 0.001, ${\upeta }_{p}^{2}$ = 0.26, *BF*_*10*_ = 2153. Looking further into the Cs, a significant liberal criterion shift was observed for aha anagrams as compared to words and to no-aha anagrams (Words: *t*(37) = 5.32, *p* < 0.001, *d* = 0.86, *BF*_*10*_ = 3718, and No-aha anagrams: *t*(37) = 3.35, *p* = 0.004, *d* = 0.54, *BF*_*10*_ = 17.8), with the latter two not differing from each other, *t*(37) = 0.46, *p* = 0.649, *d* = 0.07, *BF*_*01*_ = 5.19.

## Experiment 2

The purpose of Experiment 2 was to see if the importance of aha moments extended to the emergence of the revelation effect when the to-be-discovered items were extra-list items and not the to-be-recognized items themselves (cf. Westerman & Greene, 1996). That is, would participants not only believe that more familiar items might lead to more aha-moment solutions or relax their recognition-decision criteria after the pleasant affect accompanying the aha experience, but also apply carry-over effects of any persisting arousal felt during such a solution to their immediately following recognition judgments. In Dougal and Schooler’s (2007) study, extra-list item solutions did not lead to the revelation effect, which was one of the reasons why they rejected the idea that the revelation effect had the same underlying mechanism as the solving effect.

### Method

#### Participants

A total of 91 participants, again American University students (ages 18–30 years) who received extra credit in psychology courses or participated for fun, were tested until we reached our goal of 64 participants who avoided the same exclusion criteria as in Experiment 1. This experiment was conducted entirely virtually, again using Zoom and Qualtrics, and participants were tested individually. None had participated in the previous experiments. As before, a sensitivity analysis indicated that our effective sample size of 64 participants enabled 85% power to detect small to medium effects (*f* = 0.19, based on one group, three measurements, and correlation of 0.39 based on Experiment 1, Experiment 2, and Pilot Experiment 1).

#### Materials, design, and procedure

Because the revelation task would be of extra-list items, we selected 64 more words from the same sources and created anagrams of comparable difficulty to those in Experiment 1 using the same process as before. Indeed, to generalize the findings to different words, this time we had four study lists, List A, List B (from Experiment 1), List C, and List D (new study lists for Experiment 2). Each study list also included eight primacy and recency buffer items, and these were the same as in Experiment 1 and did not differ between lists. Each participant received only one study list.

There were two recognition-test lists, Test 1 and Test 2, and each participant received only one test. Test 1 comprised List A and List B words, and Test 2 comprised List C and List D words. When a participant received Test 1, the Test 2 words served as the extra-list items, either to-be-copied words or to-be-solved anagrams. Similarly, when a participant received Test 2, Test 1 words served as the extra-list items. Additionally, the words in Tests 1 and 2 were randomly yoked (within the constraint that they had equal numbers of letters), so that if a given word, say “animal” from List A, was the to-be-recognized word, another word from List C, say “washer” was always the to-be-copied or to-be-solved extra-list word that appeared before “animal,” and vice versa (“animal” was the to-be-copied or to-be-solved extra list word before a memory judgment was made on “washer”). Across eight counterbalancing groups, two separate sets of words were tested, and each word in a given list was a target or a lure, as well as a to-be-copied or to-be-solved anagram during the revelation task equally often.

The procedure was very much like that of Experiment 1. Participants studied a single list of words and then received a yes/no recognition test. This time, however, they were told that before each to-be-decided-on test word, first they would need to do a task with an unrelated and definitely not studied item. The task would be either copying this item or solving an anagram of such an item. Then they would be given the test word to make a target or lure decision on. Finally, if they had solved an anagram during that trial, they would need to indicate whether they had had an aha moment while doing so (same aha moment instructions were given as in Experiment 1).

Another deviation from the method of Experiment 1 was that participants no longer indicated whether they solved the anagram early or later. This was because in the previous experiment emergence of aha moments was not any different for later-solved anagrams. Of main interest was whether the revelation effect would again be limited to those situations when aha moments occurred, even though this time the experience would be for the solution of an unrelated, not-studied word for which no memory judgment was asked for. The to-be-recognized item that required a yes/no judgment was always in word form that never needed to be revealed.

### Results

The results are presented in Tables 3 and 4. A one-way repeated-measures ANOVA was significant, *F*(2, 126) = 13.27, *p* < 0.001, ${\upeta }_{p}^{2}$ = 0.17, *BF*_*10*_ = 2985. Post hoc tests showed that those anagrams whose solutions were accompanied by aha moments led to the revelation effect, *t*(63) = 4.37, *p* < 0.001, *d* = 0.55, *BF*_*10*_ = 428.6. Indeed, a greater number of “yes” responses were given to solved anagrams accompanied by aha moments compared to those solved without aha moments, as well, *t*(63) = 4.06, *p* < 0.001, *d* = 0.51, *BF*_*10*_ = 156.7. No revelation effect occurred with anagrams solved without aha moments; that is, responses after copying words and after solving non-aha anagrams were not different from each other, *t*(63) = 0.16, *p* = 0.876, *d* = 0.12, *BF*_*01*_ = 7.21. Thus, even though the revelation task involved solving an anagram unrelated to the to-be-recognized word, the aha experience from the solution task nevertheless seemed to carry over to the ensuing recognition judgment and lead to greater proportions of hits and false alarm responses.

**Table 3:** Mean percent “yes” responses (targets and lures combined) and counts in the word and anagram conditions in Experiment 2. Standard deviations are in parentheses

Words	Aha anagrams	Non-aha anagrams
56.2	65.1	55.9
(14.0)	(16.7)	(16.0)

**Table 4:** d's and Cs in the word and with- and without-aha-moment anagram solution conditions of Experiment 2, as well as mean hits and false alarms

	Words	Aha anagrams	No-aha anagrams
Hits	0.72	0.79	0.70
	(0.15)	(0.23)	(0.20)
FA	0.40	0.51	0.44
	(0.20)	(0.27)	(0.25)
d'	0.96	1.08	0.87
	(0.73)	(1.53)	(1.22)
C	−0.17	−0.58	−0.25
	(0.44)	(0.68)	(0.62)

As in Experiment 1, the percentage of solutions accompanied by aha moments was greater in the case of items reported as having been recognized compared to the case of items reported as not recognized (49.6% vs. 42.3%, *t*(62) = 3.06, *p* = 0.003, *d* = 0.39, *BF*_*10*_ = 9.12), corroborating the observation that the memory illusion was influenced by the aha experience. We should note that this difference was somewhat larger in Experiment 1, perhaps because the solved anagrams were themselves the to-be-recognized items, thereby increasing the influence of the aha moment on the memory decision.

In keeping with Experiment 1, the d's did not differ across conditions, *F*(1.69, 104.97) = 0.637, *p* = 0.506, ${\upeta }_{p}^{2}$ = 0.01, *BF*_*01*_ = 10.8. And, once again, the Cs showed differences, *F*(1.84, 113.87) = 13.54, *p* < 0.001, ${\upeta }_{p}^{2}$ = 0.18, *BF*_*10*_ = 3825, with a more liberal response bias for aha-moment anagrams as compared to words and no-aha-moment anagrams (Words: *t*(62) = 4.92, *p* < 0.001, *d* = 0.62, *BF*_*10*_ = 2655, No-aha-moment anagrams: *t*(62) = 3.47, *p* = 0.002, *d* = 0.55, *BF*_*10*_ = 27.5), the latter two not differing from each other, *t*(62) = 1.12, *p* = 0.266, *d* = 0.14, *BF*_*01*_ = 3.98.

## Discussion

In two experiments, we found that in a revelation effect task using anagrams, only those anagrams whose solutions were accompanied by an aha moment led to a greater number of “yes” responses during the recognition test. This was true both for when the task involved the revelation of the to-be-recognized words themselves as well as the revelation of extra-list words, known by the participants to not have been studied and thus not related to the recognition task.

Experiment 1 findings are consistent with the idea that participants may believe aha moments should be more likely in cases of more familiar items, especially because the memory decision at least overlapped with the discovery of the anagram solution, as the to-be-recognized word was the anagram solution itself. Incidental data show that participants’ aha response frequency was greater for target than for lure items (62.9% vs. 49.5%), suggesting that solving of familiar items might indeed be eliciting more aha moments; but, within just “yes” answers, these frequencies were 65.0% for targets and 60.3% for lures, suggesting that, even if familiarity can influence the emergence of aha moments, participants might be overestimating its magnitude. That is, this 13% difference between the frequencies of aha moments with target items compared to that with lure items might be considered to be consistent with the idea that familiar items can elicit more aha moments. Looking at just the “yes” answers, however (i.e., only the items that participants deemed familiar), this difference between target aha response frequencies and lure aha response frequencies was only 5%, with aha-response frequencies just a bit more than that with overall targets but considerably greater than that with overall lures, which could be attributed to the participants’ beliefs that if an item induces an aha moment, then it must have been studied. That is, even though the aha moment during the anagram solution is real, its extension to the memory decision can be false (cf. Danek & Wiley, 2017), a discrepancy that would be more evident with lure items and consistent with robust revelation effects shown even without a study phase (Frigo et al., 1999). As such, this belief component might be akin to those obtained in metacognitive studies of font-size and loudness illusions, in which bigger-font and louder-item presentations induce participants to judge them to be more likely to be remembered. These misjudgments are also thought to be both fluency-based and, more importantly, also partly due to beliefs (cf. Mueller et al., 2014; Tatz et al., 2021). Of course, in the present case, this belief might or might not be warranted because although there is evidence that problem solutions leading to aha moments are more memorable in the ensuing recognition test (e.g., Auble et al., 1979), we do not know whether the converse is also true. An interesting future direction could be to test directly whether aha moments are indeed more likely for episodically familiar items and whether participants might also rely on this belief (warranted or not) as a proxy more often when their confidence in their subjective familiarity is low (as in the case of lure items) and the aha moment experience further induces them to relax their recognition criterion.

Experiment 2 results showed that similar effects arose when the item that was solved before the memory decision was unrelated to the to-be-recognized item itself. We suggest that in such cases, even though the aha moment is strictly for the task solution itself without overlapping with the memory decision, because the experience is a pleasant one (e.g., Gick & Lockhart, 1995) and pleasant affect tends to produce more liberal decision criteria (e.g., Park & Banaji, 2000), participants were more likely to say “yes” after aha solutions. We found that only those items following an aha solution showed a more liberal response bias, and the Cs did not differ between items not needing to be solved and those that followed non-aha solutions. Of course, the same was true also in Experiment 1, and the pleasant affect following aha moments could have been the sole reason for the criterion change there, as well. However, it could also have served to reinforce the beliefs discussed above when the solved anagram was also the item to be decided on. These experiments were not designed to tease those explanations apart.

Although also not designed to specifically address theoretical explanations of the revelation effect, our results were consistent with theories that implicate criterion changes in the production of the revelation effect. However, the explanations for the criterion changes now need to also take into consideration the integral role of aha experiences. For instance, the criterion-flux hypothesis (Hockley & Niewiadomski, 2001) proposes that the revelation task overloads working memory and disrupts test-item information for that trial, which induces participants to temporarily relax their response criterion until the next trial. One might speculate that, when present, an aha moment might not only add to this disruption in terms of attention, but the surprising jolt can recalibrate participants’ criterion relaxation thresholds in comparison, thus enabling no-aha trials to lose this propensity. The decrement-to-familiarity hypothesis (Hicks & Marsh, 1998), proposes that the activation of competing responses during the revelation task makes the task more difficult and induces participants to relax their recognition response criterion as they tend to do with more difficult tasks in general (cf. Hirshman, 1995). Because task difficulty itself leads to mixed results in revelation effect studies, however, perhaps it is the aha moments that are responsible for relaxation of criteria rather than the difficulty of the task per se.

The discrepancy-attribution hypothesis (Whittlesea & Williams, 1998) proposes that after a revelation task, a to-be-recognized item is easier to process in terms of fluency compared to what went on during the task, and this comparative ease gets misattributed to memory (cf. Dougal & Schooler, 2007). Then the question becomes why tasks ending with aha solutions would lead to less discrepancy in terms of processing fluency. That is, an aha moment would have cut off any remaining analytical or trial and error way of solving that, and if it had gone on, would have meant even less fluency during the solving process, and thus analytically solved items should have had more discrepancy. Writing in the aha solutions would be more akin to copying of regular words, thus leading to greater perceived fluency. To this end, Whittlesea and Williams propose a more nuanced interpretation in that it’s not just fluency per se, but “surprising fluency” that leads to the discrepancy-(mis)attribution. It is conceivable then that such surprising fluency increases both the chances of an aha moment and an unrelated positivity bias at the same time, but at this point we have no evidence for it. To the extent that the surprise element is necessary, we speculate that thinking of surprise as a separate but integral part of the aha moment would be more parsimonious. We thus do agree that there is misattribution, but we do not believe it is the surprising ease of processing per se or fluency that is misattributed to memory but rather the phenomenological state of the aha moment, and also it is not the discrimination per se that is affected but the criterion of acceptance.

Such a view would be more in line with a belief that discovering episodically familiar items is more likely to cause aha moments as well as the rewarding effects of aha moments, both instances leading to relaxing of criteria (cf. Shen et al., 2016). Indeed, this view would also be consistent with the relatively newly proposed Eureka Heuristic model in decision making (Laukkonen et al., 2023), which holds that aha moments promote a sense of confidence and even unrelated aha experiences make facts seem truer. Aha experiences are said to be metacognitive feeling heuristics, and visceral changes during these moments act as cues for gauging the trustworthiness of decisions (e.g., Fernández Velasco & Loev, 2024). In our case, this heuristic elicited during the anagram solution task could have manifested itself in the ensuing episodic recognition decision.

One general limitation of our study is that we cannot tell the exact relationship between aha moments and the revelation effect. It could be that it was the recognition decision that may have impacted whether participants reported that they experienced an aha moment during the anagram solution. One way of alleviating this directional ambiguity would have been to reverse the order of responding for the recognition and aha questions for half of the items, especially in Experiment 2. Given that participants were always aware that both questions would be asked in each case anyway, we had thought that we could not really tease out the impact of recognition on aha moment decisions regardless of the response order. Thus, we decided to minimize the contamination of recognition decisions, as they were the critical responses that would determine whether the revelation effect emerged or not. Hence, we put the recognition decision first, which should also have worked more “against” our hypothesis that aha moments would affect recognition, and by not switching orders, perhaps also drawn less attention to the purpose of the study. In hindsight, however, in Experiment 2, because our argument for the emergence of revelation effect relies on carry-over effects (with aha responses being related to the extra-list item solutions and thus ostensibly decoupled from recognition responses), testing directionality by switching recognition and aha response orders might have been informative. For instance, in those cases, if the aha response came *before* the recognition response, participants might have been more likely to separate what happens during the solving task from the following recognition task and reduced the carry-over effects of aha moments. A future study could include recognition responses, with only some of the anagram solutions followed by asking about aha decisions and also switching the order of aha and recognition questions.

Another issue is how we might reconcile the idea of an aha moment with some simple or motor revelation tasks that seem unlikely to produce insight (cf. Aßfalg et al., 2017b; indeed Bernstein, et al., 2009, showed that even a successful solution on the revelation task may not really be necessary). One possibility might be that because those studies did not ask for any aha moment judgments, aha moments cannot be ruled out. For instance, perhaps in those cases, aha moments could still have occurred for some items but might have been attached instead to the act of remembering rather than being a carry-over effect from the revelation task. Or, as suggested for non-episodic decisions in the present *Introduction*, a given revelation task may affect recognition decisions at different levels or via different mechanisms, as well, and when the possibility of aha moments enters the procedure explicitly, it becomes the most salient variable and dampens the effectiveness of other levels/mechanisms. At this point, we can only speculate.

Perhaps related to the above issue is how we have been able to show the revelation effect previously in similar anagram studies when a solution has almost always been provided to the participants to ensure discovery of the intended word. In these studies, to make discovery a relatively simple and uniform task, experimenters often used the same algorithm to work for all solutions, and little attention was paid to whether the participants were solving each anagram on their own or making use of the algorithm. It is possible then that some anagrams were self-solved anyway with an accompanying aha experience, and even when the algorithm was needed, the switching of letters in the same order every time could have become automatic enough at some point for participants to make use of it only partially. In our first pilot experiment, when we did have the algorithm condition, there were too many different algorithms for the participants to be able to even partially memorize. Thus, although we did not ask for an aha moment indication, if an algorithm had to be resorted to, it was likely to have been used fully or nearly fully, making an aha moment comparatively unlikely during the solution.

Also, just because we did not see a revelation effect with no-aha solutions does not mean that we can conclude it cannot emerge in the absence of an aha moment. In this study, we simply showed that when aha moments were made salient, any revelation effect with the same manipulation but without the aha moment did not reach significance. A future direction might be to see if an aha moment (for the solution of the revelation task or even the memory process itself) could be considered a mediator of the revelation effect in recognition memory or is yet another manipulated task variable that can be overpowering but also interacts with other variables in producing the revelation effect. For instance, tracking aha moments while manipulating other variables that elicit the revelation effect (e.g., rotating letters or finding synonyms) or variables that influence its magnitude (e.g., the similarity between lures and targets) could inform whether aha moments, irrespective of other manipulations, are the main contributor of the effect or can even erase some of the effects of the other manipulations. This would be consistent with a study by Laukkonen et al. (2020) in which artificially induced aha moments (via anagram tasks) were found to bias the participants to judge accompanying ideas or facts as true. We could also see whether some of the boundary conditions, such as the limitation of the recognition revelation effect to episodic memory, could be eliminated when we look at only aha moments.

Another future direction might be to explore what exactly it is in the phenomenological state of the aha experience that elicits the revelation effect. More specifically, whether it is indeed the arousal associated with the aha experience, its pleasant nature, or an inflated sense of confidence unrelated to the emotional arousal that leads to the adoption of a more liberal response criterion (cf. Moroshkina et al., 2024). To this end, one could explore the effects of pure arousal with or without the aha moment (cf. Fiacconi et al., 2023; Goldinger & Hansen, 2005), for instance by including auditory stimulation (e.g., rousing music) during the testing of some items and also seeing how confidence ratings would map onto the responses. Of course, a more direct way would need to entail EEG or other physiological data as well (cf. Oh et al., 2020). In sum, although at this point we do not know exactly how, it is clear that an aha experience (albeit a self-reported one), in the present case associated with a revelation task, seems to play a crucial role in the emergence of the revelation effect in episodic recognition decisions.

## Open Practices Statement

None of the experiments were preregistered. Finalized materials for both experiments, as well as the more detailed methods and results of the pilot experiments, are presented in the Online Supplementary Materials. Because IRB approval was for publicly publishing of aggregate data only, we are unable to make individual participant data available online. However, they can be obtained on request from the first or the third author.

## Supplementary Information

Below is the link to the electronic supplementary material.

Supplementary file1 (DOCX 21 kb)

Supplementary file2 (DOCX 16 kb)

## Data Availability

See Open Practices Statement.

## Footnotes

1 “…If you successfully solved the anagram in the first step, you indicate whether you had any type of an Aha Moment while solving it. That is, did the solution hit you suddenly (aha!) or did the combinations you tried simply end up making that word after strategic effort. If the solution jumps at you at any point, and makes you realize “oh that’s it,” rather than “ho hum, there’s the word,” then it’s an “aha.” That is, if getting that solution excites you (surprises you or makes you proud and includes any kind of emotion) it is an “aha” solution…Before moving on, let’s clarify what an “aha!” insight is. Can you explain your understanding of it? Can you give an example?”.

2 Even though we used confidence ratings as in the pilot experiments (which could have given us weighted scores as a more sensitive measure of memory if the Yes/No answers had not been robust enough), we are not reporting them because the Yes/No answers yielded clear memory results on their own, and thus the weighted score results would have been redundant.

3 ^3^ When solved anagrams were further divided into the early and later solved categories, some participants did not always have both aha and non-aha instances. Hence the differences in degrees of freedom. Five participants were excluded from the early-solved comparisons and six participants were excluded from the later-solved comparisons. These participants were excluded from these specific analyses but retained in the main ANOVA when we collapsed the categories.
