# Supplementary material for: The role of revelation in the revelation effect in episodic recognition
Source: Mem Cognit. 2026 May 28;54(4):1091–101. doi: 10.3758/s13421-025-01756-3 (PMC13253584; doi:10.3758/s13421-025-01756-3)
Supplement: Supplementary file 1 — Supplementary file1 (DOCX 21 kb) [file 13421_2025_1756_MOESM1_ESM.docx]

SUPPLEMENTARY MATERIALS 1

**Pilot Experiments**

**Methods**

**Participants**. Both pilot experiments were in-person, and participants (American University students 18-30 years) were tested individually or in small groups and received extra credit in various psychology courses. For the first experiment, 97 participants were tested until we reached our goal of 48 participants, and for the second experiment, 58 participants were tested until we reached our goal of 32 participants who avoided the exclusion criteria. The exclusion criteria were: 1) not following instructions (e.g., skipping of copying words or of writing in anagram solutions) 2) not being able to solve at least 80% of the anagrams. For the first pilot experiment, there was also an exclusion criterion based on not being able to solve at least 60% of the anagrams without an algorithm.

**Materials, Design, and Procedure.**

***Pilot Experiment 1****.* Single-solution anagrams, always with the first letter given, were constructed from 88 5-7 letter words (Gilhooly, 1978; Rawlinson, 1976). For this experiment, we had easy and difficult versions such as PLAESE and PSELEA for PLEASE (following the algorithms 123546 and 153264, respectively). There were 12 different possible algorithms to prevent memorization so that if participants could not solve an anagram, they would need to look at its solution key rather than look at it partially or learn to rearrange the letters automatically. Eight words were used as primacy and recency buffers (four each) during presentation and also as practice items (for solving anagrams) before the recognition test. The remaining 80 words were divided randomly into two sets of 40 words each, within the constraint that a given word in a list had a yoked word of the same length and started with the same letter in the other list. Words in one set acted as the target items and those in the other as the lure items for one half of the participants, and vice versa for the other half of the participants.

During presentation, words were printed on a single sheet of paper in two columns, and participants uncovered each word at 2-second intervals by sliding a blank sheet of paper when prompted by the experimenter. Participants were told to simply try to remember each word. During the recognition test, participants were given all of the 80 items (40 targets and 40 lures), with half of each of targets and lures (20) presented as words and the other half as anagrams (10 easy and 10 difficult). Across 8 counterbalancing groups, each word appeared equally often as a target or a lure, as a word or an anagram, and as an easy or a difficult anagram in an all repeated-measures design.

During the yes/no recognition test, items were presented in a booklet form, with each item occupying a single line. Participants were told that they would sometimes see a word and copy it in the fragment next to it (e.g., PLEASE; P - - - - -) and then make a Yes/No judgment on whether the word had been included in the study list (and also give a confidence rating between 1 (guess) and 3 (sure)—although we did not analyze these ratings). And sometimes the word would be disguised in the form of an anagram (and these would be asterisked), and they would need to write the solution in the fragment next to it before making the recognition judgment (e.g., *PSELEA; P - - - - -). They were given up to 30 s to copy words or solve and write-in words. If they were unsuccessful in solving an anagram during that time, they were to look at the algorithm for that word at the far end of the paper in the last column and then write in the word, and it was made clear that this was the last resort. After the copying/solving of each word and the recognition judgment, if participants had indeed solved an anagram, they were asked to put a check mark under one of three categories specified in the column right after the recognition judgment column: 1) Immediate (first 2-3 seconds); 2) Later (next 27-28 seconds); or 3) Algorithm (used key). They then moved on to the next word on the recognition list.

***Pilot Experiment 2****.* The materials, design, and procedure were largely the same, but, informed by the results of the first pilot experiment, some changes were made. First, only some of the same materials were selected based on the subjective difficulty of their anagram-version solutions (not too difficult and not too easy), thus taking out the easy/difficult anagram condition as a factor. Second, the numbers of targets and lures were shortened to 32 each. And finally, because the main purpose of this study was to see if aha moment indications would mirror the algorithm condition results, there was no algorithm condition—if the participants could not solve an anagram, they simply left it blank, but for solved anagrams, after giving the recognition judgment and indicating whether the solution was obtained immediately or later, they moved to the last column where they indicated whether the solution had come accompanied by an aha moment or not.

**Results.**  All results are shown in Table S1. Responses to targets and lures were combined for analyses. In the first pilot experiment, there emerged no differences between easy and difficult anagrams in terms of the revelation effect, *t* < 1. Also, although more of the difficult anagrams tended to be solved later, it wasn’t always the case. Thus, we instead focused only on each participant’s own immediate/later indications, which we thought would be a better gauge of personal difficulty and would thus better preserve individual differences in solution prowess while keeping any effects of aha moments the same.

As can be seen, the revelation effect was quite robust with self-solved anagrams, *t*(47) = 8.49, *p* < .001, *d* = 1.23, *BF_10_* = 25.0, although there were no differences as a function of the immediately- and later-solved anagrams, *t* < 1, *BF_01_* = 6.25^[[1]](#footnote-1)^. Most importantly for present purposes, no revelation effect emerged with algorithm-solved anagrams, *t* < 1, *BF_01_* = 6.38. The revelation effect was confined to self-solved anagrams.

In the second pilot experiment, overall, there was no revelation effect, and there was at least moderate evidence that anagram solutions were not given more “yes” responses than were copied words, *t* < 1, *BF_01_* = 4.87. However, when we analyzed only those anagrams that had been accompanied by an aha experience separately, the revelation effect did emerge, *t*(31) = 1.98, *p* = .028, *d* = .35, *BF_10_* = 1.06 (although note that *BF* was inconclusive). Because the effect was absent even overall (when aha and non-aha responses were combined), we did not analyze non-aha responses separately. Interestingly, this time, the effect seemed to have, if anything, been carried more by immediately- rather than later-solved anagrams, although the difference between the two conditions did not reach significance. But because these results were a bit different from those of the previous experiment, we kept the immediate/later variable in the first main experiment. Perhaps differences would emerge when we had power to detect them, or maybe an aha moment could happen both when a word pops out of an anagram immediately as well as after an impasse.

**Table S1**. Mean Percent “Yes” Responses (targets and lures combined) in Word and Anagram Conditions in the Pilot Experiments. Standard deviations are in parentheses. Note that due to unsolved anagrams, the number of observations was not equal in all conditions.

__________________________________________________________________________

Word Anagram

________________________________________________________________

Immediate Later Immediate/Later Algorithm

Experiment 1 **50.6** 63.7 63.1 **63.7 50.6**

**(11.0)** (15.5) (20.3) **(14.0)** **(25.1)**

Experiment 2 Overall (aha + nonaha) Aha Only_____

Imm. Later Imm./Later Imm. Later Imm./Later

**54.5** 64.0 45.7 **56.1** 72.3 56.1 **64.0**

**(16.4)** (26.0) (25.4) **(15.7)** (30.8) (32.0) **(24.7)**

____________________________________________________________________________

1. A one-way repeated-measures ANOVA was significant, *F*(2.20, 103.18) = 10.32, p < .001, $\eta_{p}^{2}$ = .18, *BF_10_* = 6168 and post-hoc tests (with Bonferroni corrections) showed that there was a revelation effect with both immediately-solved and with later-solved anagrams when compared with normal words, *ts*(47) = 4.02 and 3.82, respectively, *ps* < .001, *BF_10_s* > 1196. [↑](#footnote-ref-1)
