# Supplementary material for: The role of revelation in the revelation effect in episodic recognition
Source: Mem Cognit. 2026 May 28;54(4):1091–101. doi: 10.3758/s13421-025-01756-3 (PMC13253584; doi:10.3758/s13421-025-01756-3)
Supplement: Supplementary file 2 — Supplementary file2 (DOCX 16 kb) [file 13421_2025_1756_MOESM2_ESM.docx]

**Materials**

Experiment 1

Study List 1 (for Groups 1A and 1B) Study List 2 (for Groups 2A and 2B)

Supreme, Table, Priest, Float Supreme, Table, Priest, Float

Bolster (Boslter) Battle (Btalte)

Melon (Mnleo) Olive (Ovlei)

Animal (Anmail) Parent (Peanrt)

Intrude (Inudert) Brute (Beurt)

Teach (Tceha) Peach (Pheac)

Kitchen (Khtniec) Naked (Neadk)

Mundane (Mudanen) Ocean (Oneca)

Umpire (Umriep) Loyal (Laoly)

Rowdy (Rwody) Leader (Ldeear)

Leash (Lhaes) Gifted (Gefidt)

Blame (Blaem) Seaside (Sesaide)

Builder (Buidler) Score (Srceo)

Eager (Ergae) Brigade (Bragide)

Ranger (Rgaenr) March (Mcahr)

Ordeal (Oadrle) Lovely (Leovly)

Nowhere (Nhoewer) Grudge (Gudgre)

Listen (Ltiesn) Sunlit (Silnut)

Staple (Sptlae) Citadel (Caidtle)

Neither (Nhireet) Mango (Manog)

Height (Hhietg) Attic (Aitct)

Scalp (Spacl) Sauce (Scaeu)

Course (Crosue) Dagger (Dgaegr)

Rider (Rrdie) Respect (Rpeestc)

Miracle (Maicrel) Maple (Mapel)

Throb (Tbrho) Wrong (Wnrgo)

Drape (Depra) Throne (Torhen)

Please (Pelase) Artist (Astirt)

Reason (Rseoan) Shrub (Srubh)

Sausage (Saueags) General (Geranel)

Flesh (Fslhe) Recital (Rectial)

Phrase (Pheras) Wrestle (Wesrtle)

Minute (Muitne) Option (Otipon)

Talent Reform Mania Printer Talent Reform Mania Printer

Test List: Combination of the two study lists.

Experiment 2

Two more study lists added to those of Experiment 1.

List 3 List 4

Supreme, Table, Priest, Float Supreme, Table, Priest, Float

Washer (Wahser) Earthen (Erathen)

Discern (Dicesrn) Various (Viarosu)

Caution (Cautino) Grave (Garev)

However (Hvwroee) Medic (Miced)

Certify (Cteiryf) Night (Nhitg)

Rifle (Refil) Anoint (Aoinnt)

Large (Lerag) Dresser (Desrrse)

Restful (Rulfest) Region (Regino)

Pierce (Peicre) Master (Mtsaer)

Study (Sutyd) Silence (Scilene)

Remain (Ramnie) Serpent (Seprtne)

Planter (Palnter) Marble (Mblrae)

Raise (Riaes) Ridge (Rdige)

Roost (Rosto) Hatred (Hrteda)

Bustle (Bestlu) Diaper (Dirpea)

Stare (Sarte) Right (Rghit)

Banjo (Bnoja) Brick (Birkc)

Album (Ablum) Chord (Crodh)

Recall (Racell) Desire (Direse)

Tenure (Teenru) Ginger (Gginer)

Clown (Colwn) Fancy (Facyn)

Force (Feroc) Guilt (Gtlui)

Desire (Deirse) Recall (Rallce)

Reward (Rawedr) Ignore (Irogne)

Honey (Heyno) Judge (Jgdue)

Logic (Lciog) Orbit (Obtri)

Rescue (Ruesce) Sister (Ssrtie)

Pilot (Ploit) Tempo (Topme)

Escort (Erstoc) Trance (Tarcne)

Travel (Tavelr) Rhyme (Rymhe)

Snack (Sanck) Porch (Proch)

Medal (Malde) Lunch (Lcuhn)

Index (Ixden) Doubt (Dubot)

Talent Reform Mania Printer Talent Reform Mania Printer

Two Test Lists: Participants receive only one, and the words/anagrams of the other serve as the extra-list items (to be copied or solved).
